# 22q11.2 duplication syndrome: elevated rate of autism spectrum disorder and need for medical screening

**DOI:** 10.1186/s13229-016-0090-z

**Published:** 2016-05-06

**Authors:** Tara L. Wenger, Judith S. Miller, Lauren M. DePolo, Ashley B. de Marchena, Caitlin C. Clements, Beverly S. Emanuel, Elaine H. Zackai, Donna M. McDonald-McGinn, Robert T. Schultz

**Affiliations:** Department of Pediatrics, Seattle Children’s Hospital, M/S OB.9.520, 4800 Sand Point Way NE, Seattle, WA USA; Center for Autism Research, Children’s Hospital of Philadelphia, 3535 Market Street, Philadelphia, PA 19104 USA; Department of Psychology, University of Pennsylvania, 3720 Walnut Street, Philadelphia, PA 19104 USA; Division of Human Genetics and Molecular Biology, Children’s Hospital of Philadelphia, 3401 Civic Center Boulevard, Philadelphia, PA 19104 USA; 22q and You Center, Children’s Hospital of Philadelphia, 3401 Civic Center Boulevard, Philadelphia, PA 19104 USA; Department of Pediatrics, University of Pennsylvania, 3401 Civic Center Boulevard, Philadelphia, PA 19104 USA

**Keywords:** 22q11.2 duplication syndrome, 22q11.2 deletion syndrome, Autism spectrum disorder, Developmental delay, Medical characterization, Medical screening, Neuropsychiatric functioning, Syndromic autism, Typically developing controls

## Abstract

**Background:**

Widespread use of microarray technology has led to increasing identification of 22q11.2 duplication syndrome (22q11.2DupS), the reciprocal syndrome of the well-characterized 22q11.2 deletion syndrome (22q11.2DS). Individuals with 22q11.2DS have elevated rates of community diagnoses of autism spectrum disorder (ASD), schizophrenia, and a range of medical problems and birth defects that necessitate extensive medical screening. Case reports of 22q11.2DupS include patients with ASD, fewer medical problems, and no schizophrenia; however, no prospective cohort study has been reported. The goals of the study were to (1) characterize the neuropsychiatric functioning of a cohort of individuals with 22q11.2DupS in comparison to large samples of typically developing controls (TDCs), ASD and 22q11.2DS; (2) estimate the prevalence of ASD in 22q11.2DupS; (3) determine whether the indications that prompted the genetic testing in 22q11.2DupS differ from 22q11.2DS and (4) determine whether comprehensive medical screening should be recommended for those diagnosed with 22q11.2DupS.

**Methods:**

Medical characterization was done by parental questionnaire and medical chart review of individuals with 22q11.2DupS (*n* = 37) and 22q11.2DS (*n* = 101). Neuropsychiatric characterization of children with 22.11.2DupS, 22q11.2DS, TDCs, and ASD was done by parent-report questionnaires; in addition, the ASD and 22q11.2DupS groups received the Autism Diagnostic Interview-Revised and Autism Diagnostic Observation Schedule.

**Results:**

Individuals with 22q11.2DupS, 22q11.2DS, and ASD had significantly impaired social interaction and adaptive behavior skills compared to TDCs. Overall, 38 % of children aged 2–18 with 22q11.2DupS had community diagnoses of ASD, but fewer (14–25 %) met on the basis of best clinical judgment that included ADI-R and ADOS data. Indications for genetic testing were significantly different for 22q11.2DupS and 22q11.2DS, with the deletions more commonly tested because of birth defects or medical problems, and the duplications because of developmental delay. However, when the screening protocol for 22q11.2DS was applied to the 22q11.2DupS sample, several medical problems were identified that would pose significant risk if left undetected.

**Conclusions:**

22q11.2DupS has a high rate of ASD at 14–25 %, among the highest of any genetic disorder. Prospective medical screening should be done for all patients with 22q11.2DupS, including those diagnosed due to developmental delays and ASD alone.

**Electronic supplementary material:**

The online version of this article (doi:10.1186/s13229-016-0090-z) contains supplementary material, which is available to authorized users.

## Background

22q11.2 deletion syndrome (22q11.2DS) is the most common and one of the best-characterized microdeletion syndromes. Individuals with 22q11.2DS have increased rates of birth defects (e.g., congenital heart disease, anomalies of the palate, limbs, spine, kidneys), medical problems (e.g., hearing loss, immune dysfunction, hypocalcemia) and neuropsychiatric issues (e.g., developmental delay, risk for psychosis) [1–5]. There are far fewer reported cases of 22q11.2 Duplication Syndrome (22q11.2DupS), the reciprocal duplication syndrome involving the exact same set of genes. Rates of 22q11.2DupS identification are clearly increasing due to the widespread use of microarray testing; however, our current understanding of the 22q11.2DupS medical and psychological phenotype remains quite limited. While there have been some published case descriptions including some case series [6], there has not yet been any systematic, protocol-driven research on the duplication disorder. Thus, clinical guidelines for management of patients with 22q11.2DupS do not exist. Moreover, the utility for psychiatric research of those with this duplication syndrome is unknown.

Prospective studies of large cohorts of individuals with 22q11.2DS suggest that about 30 % of individuals with 22q11.2DS have attention-deficit/hyperactivity disorder [3] and roughly 25 % develop psychosis [4]. There are also reports of an increase in features of autism spectrum disorder (ASD) in this population, with estimated rates of ASD diagnoses in clinically ascertained samples ranging from 10–40 % [3, 7–9]. However, data from gold standard, in-person diagnostic measures indicate that a much lower percentage of children truly fulfill the necessary criteria for an ASD diagnosis [5, 10], which include both social-communication deficits and repetitive/stereotyped behaviors. Some children with 22q11.2DS exhibit the social communication symptoms of ASD—deficits in social-emotional reciprocity, nonverbal communication (e.g., pointing or nodding), and developing relationships [3]. However, rates of repetitive/stereotyped behaviors are low in the 22q11.2DS samples studied to date, thus resulting in few individuals meeting full diagnostic criteria for ASD.

Although there are no research cohort studies to date, case series of children with 22q11.2DupS have revealed a wide range of neuropsychiatric functioning, including some cases with ASD, but no reported cases of psychosis. In fact, Rees and colleagues [6] suggested that 22q11.2DupS might be protective against the development of schizophrenia. However, a limitation of prior studies of 22q11.2DupS is the absence of carefully measured neuropsychiatric dimensions using research-validated questionnaires and/or direct clinician assessment using gold standard research procedures.

The current study utilized medical chart review, parent-report questionnaires and, in a subset of our sample of youth with 22q11.2DupS, direct clinical assessment for an ASD diagnosis. The goals of the study included the following: (1) to characterize the neuropsychiatric and adaptive functioning profile of individuals with 22q11.2DupS and (2) to determine the prevalence of ASD in our sample with 22q11.2DupS. Both of these goals were accomplished by comparing patients with 22q11.2DupS to age- and sex-matched patients with 22q11.2 DS, idiopathic ASD, and typically developing individuals. In addition, the study sought to (3) determine whether the indications that prompted the genetic testing in 22q11.2DupS differ from 22q11.2DS and (4) determine whether the comprehensive medical screening recommended in 22q11.2DS should be applied to those diagnosed with 22q11.2DupS.

## Methods

### Identification of individuals with 22q11.2 syndromes

**Fig. 1:**
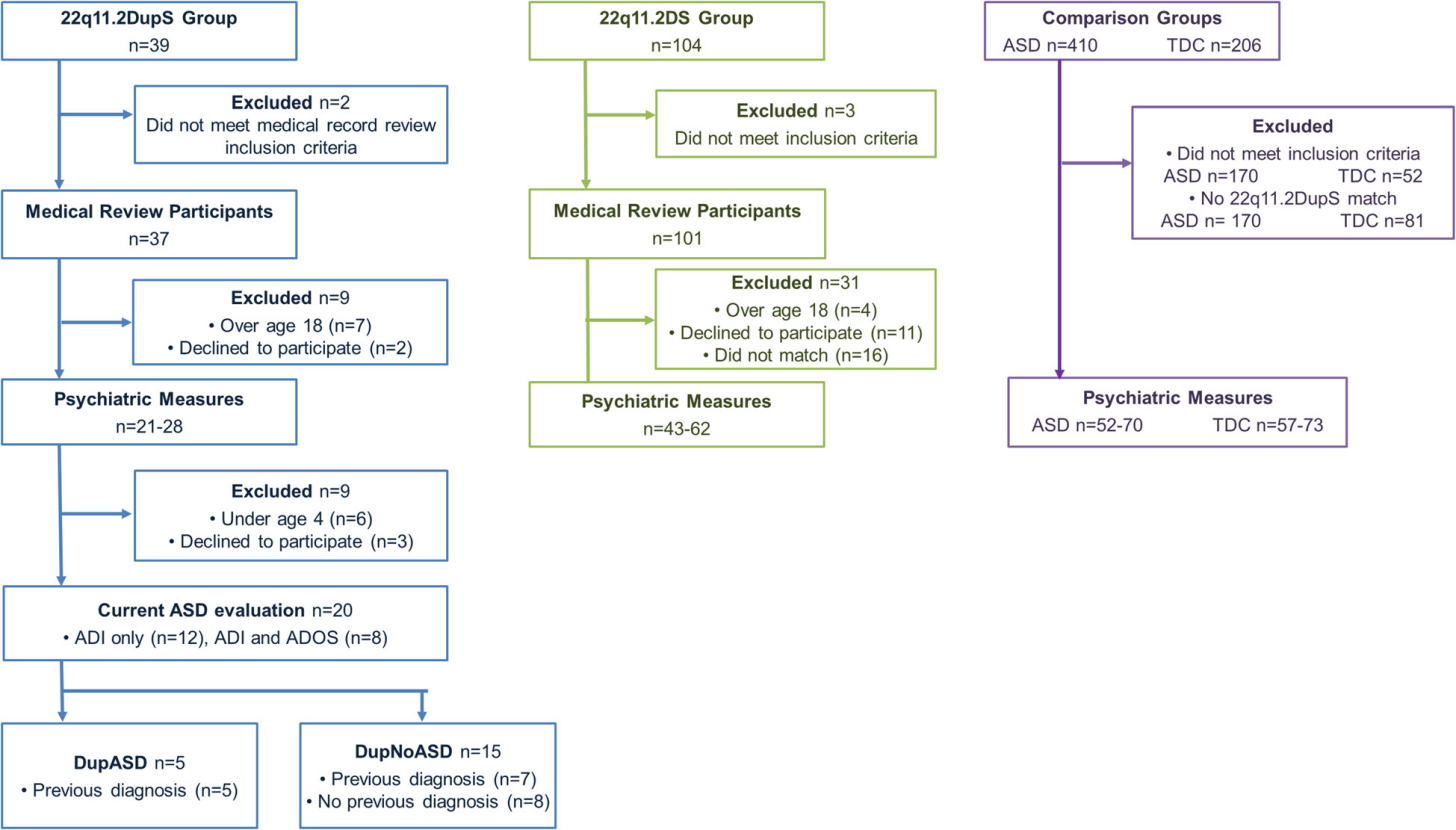
Consort diagram for enrollment, participation, evaluation, and analysis. The diagram details enrollment, participation, evaluation, and analysis for 22q11.2DupS (left), 22q11.2DS (middle), and ASD and TDC (*right*) participants at each stage of the current study. Abbreviations: *ADI-R* Autism Diagnostic Interview-Revised, *ADOS* Autism Diagnostic Observation Schedule, *ASD* autism spectrum disorder, *TDC* typically developing children

Participants were drawn from the clinical population of patients with 22q11.2DupS (age 2.9–46.7 years, *n* = 39; see Fig. 1 for enrollment information) who had received specialty clinical care at The Children’s Hospital of Philadelphia (CHOP). As microarray testing is performed in the CHOP cytogenetics laboratory, referrals to the “22q and You” clinic were made for children who had identification of 22q11.2DupS or 22q11.2DS for any referral indication. Children who received microarrays because of developmental or medical concerns all went through the same clinical laboratory and had equal opportunity for identification. Medical record review was conducted on 37 patients (two were excluded—see below). Age- and sex-matched patients with 22q11.2DS were selected from a cohort of deletion patients (age 2.0–51.2 years, *n* = 104) already enrolled in research at CHOP. All participants in the 22q11.2DupS and 22q11.2DS groups had a typical (LCR-A to LCR-D) duplication or deletion as confirmed with clinical or research testing via SNP microarray or multiplex ligation probe amplification.

Five participants (*n* = 2 with duplications, *n* = 3 with deletions) were excluded for the following reasons: (1) presence of a second genetic disorder that would be expected to affect medical or psychological health (e.g., mitochondrial disorder, X-linked chronic granulomatous disease, or severe combined immunodeficiency requiring bone marrow transplant); (2) children under age 18 years living in foster care since parental questionnaires were used; and (3) extreme prematurity (gestational age of less than 30 weeks). Informed consent for participation was obtained for all participants; these documents and procedures had been approved by CHOP’s Institutional Review Board (Protocol #13-101307).

### Characterization of autism spectrum disorder in individuals with 22q11.2DupS

#### Participants

Patients with 22q11.2DupS between the ages of 4 and 17 years were offered a comprehensive diagnostic evaluation for ASD, and 20 out of 24 chose to participate (see Fig. 1). For these families, an expert psychologist with extensive ASD experience administered the Autism Diagnostic Interview-Revised (ADI-R) [11], a gold standard autism evaluation instrument, via phone. A subset (*n* = 8) of the 20 participants then completed an in-person evaluation with the Autism Diagnostic Observation Schedule (ADOS) [12]. Some parents who were willing to bring their child for in-person evaluation had prior concern about ASD.

#### Measures

Diagnosis was based on the cumulative information across all measures, but relied in particular on two gold standard instruments—the ADI-R and the ADOS. The ADI-R is a standardized, semi-structured interview with the participant’s parent or primary caregiver that assesses the quality of reciprocal social interactions, language, and communication skills, and restricted and repetitive interests and behaviors. This ADI-R provides information about both the participant’s current functioning and their developmental history, which is particularly important for establishing a historical ASD diagnosis in older participants. The ADI-R be administered in-person or by phone. The ADOS is completed in-person through a series of semi-structured activities with a clinician who is an expert in making ASD diagnoses; it requires extensive training to administer. The examiner stages a series of interactions with the participant in order to assess the social communication ability and the presence or absence of restricted and repetitive behaviors. Parents of participants provided relevant psychoeducational materials to supplement their children’s charts already on file at CHOP, including prior developmental assessments and school records (e.g., individualized education plans).

#### Diagnostic decision-making

Final research diagnoses for this study were based DSM-5 ASD diagnostic criteria. Two clinical psychologists with expertise in ASD reviewed all information (i.e., information obtained from medical and educational chart review, ADI-R, and ADOS, when available) and completed a DSM-5 checklist for each participant. Per DSM-5, participants who met criteria for 3 of 3 social-communication symptoms and 2 of 4 restricted and repetitive behavior symptoms were given a research diagnosis of ASD (Fig. 1).

### Characterization of neuropsychiatric and adaptive functioning profiles in individuals with 22q11.2DupS

#### Participants

Comparison groups comprised of (1) children with idiopathic ASD, (2) typically developing children (TDCs), and (3) youth with 22q11.2 DS. Each of the comparison groups was matched on age and sex to the 22q11.2DupS cohort. ASD and TDC data came from the Center for Autism Research database and included all of the same questionnaires and in-person measures given to the 22q11.2DupS group. The 22q11.2 DS were recruited from CHOP’s specialty “22q and You” clinic and were administered all of the same questionnaires. Children with idiopathic ASD or typical development were excluded if there was evidence of an independent medical event (e.g., head injury, significant drug exposure during pregnancy), comorbid medical problem suggesting an underlying genetic syndrome (e.g., multiple birth defects without explanation, dysmorphic features), or clinically significant copy number variant on clinical or research SNP array (available for nearly every ASD participant). Exclusion was determined from review of three medical and neurodevelopmental history questionnaires by the same physician who reviewed the 22q11.2DupS and 22q11.2 charts as will be described below (Fig. 1). Of note, one child recruited as idiopathic ASD was later identified on research microarray testing to have 22q11.2DupS and was moved into that group for analysis.

Recruitment followed a 3:1 matching strategy to form our groups and to enhance statistical power. Each 22q11.2DupS participant (*n* = 28) was matched on age and sex to three eligible children from each comparison group (ASD, TDC, and 22q11.2DS), with 1:1 matching for participants younger than 4 years since the comparison group sample sizes were smaller in these youngest ages. Age matching was within 3 years, with the average deviation of ± ~0.5 years of age (Table 1). Across measures, the final sample averaged about 2.5 participants in each comparison group for each participant with 22q11.2DupS.

**Table 1:** Group demographics

	Medical chart review			Psychiatric and behavioral Instruments																	
				SRS			SCQ			Vineland-II			CASI-4R			ADI-R			ADOS		
	n	Age M (SD)	% male	*n*	Age M (SD)	% male	*n*	Age M (SD)	% male	*n*	Age M (SD)	% male	*n*	Age M (SD)	% male	*n*	Age M (SD)	% male	*n*	Age M (SD)	% male
ASD	–	–	–	68	7.8 (3.4)	80.9	56	9.0 (2.7)	76.8	55	9.0 (2.7)	76.4	52	9.0 (2.7)	75.0	70	7.8 (3.5)	80.0	70	7.8 (3.5)	80.0
22q11.2DupS	37	14.0 (13.5)	75.7	28	7.1 (3.4)	78.8	21	8.4 (3.0)	81.0	27	7.2 (3.5)	77.8	21	8.4 (3.0)	81.0	20	8.2 (3.2)	80.0	8	8.3 (2.9)	87.5
22q11.2DS	101	8.9 (7.9)	59.4	62	7.6 (3.8)	75.8	53	8.7 (3.5)	77.4	60	7.4 (3.9)	75.0	43	8.8 (3.0)	72.1	–	–	–	–	–	–
TDC	–	–	–	73	7.8 (3.5)	76.7	57	9.3 (2.4)	78.9	57	9.3 (2.4)	78.9	57	9.3 (2.4)	78.9	–	–	–	–	–	–

Children with 22q11.2DS, idiopathic ASD, or TDC were matched on age and sex to the 22q11.2DupS cohort. Each questionnaire was completed on-line. Because the allowable age range differs for each questionnaire, sample sizes vary somewhat across the different study instruments

*Abbreviations*: *ASD* autism spectrum disorder, *CASI-4R* Child and Adolescent Symptom Inventory-4R, *SCQ* Social Communication Questionnaire, *SRS* Social Responsiveness Scale, *TDC* typically developing controls, *Vineland-II* Vineland Adaptive Behavior Scales-II

Parents of participants in all four cohorts completed neuropsychiatric and adaptive functioning questionnaires. Completion rates were very high (96.4 % for 22q11.2DupS and 88.6 % for 22q11.2DS completion). Each questionnaire was sent only to participants whose age fell within the age range validated for that questionnaire; because the age range differs for each questionnaire, sample sizes vary somewhat across the different study instruments but all analyses included at least twice as many comparison group participants as those with 22q11.2DupS (see Table 1 and Fig. 1). There were no significant group differences for age or sex for any test instrument (Table 1).

#### Questionnaires

1. Social Communication Questionnaire, Lifetime (SCQ) [13]—Ages 4–18+ years. This parent-report tool is widely used in autism research due to its brief nature (40-items) and significant item-level parallels to the Autism Diagnostic Interview-Revised.
2. Social Responsiveness Scale (SRS) [14]—Ages 4–18 years and SRS-2 [15]—Ages 2.5–4.5 years. This parent-report questionnaire measures current levels of social responsiveness.
3. Vineland Adaptive Behavior Scales, Second edition, Parent/Caregiver Form (Vineland-II) [16]—Ages 0–18 years. The Vineland-II measures adaptive functioning in the areas of Communication, Daily Living Skills, and Socialization, and a total Adaptive Behavior Composite (Composite) widely used in the assessment of children with and without medical and/or psychiatric disorders.
4. Child and Adolescent Symptom Inventory-4R (CASI-4R) [17]—Ages 5–18 years, depending on subscale. This parent-report questionnaire is based on DSM-IV checklists for different psychiatric disorders, including, anxiety, disruptive behavior, psychosis, mood, ASD, and ADHD. The CASI-4R provides symptom severity *T*-scores from a normative dataset.

### Analyses

The four groups were compared on each questionnaire using an analysis of variance (ANOVA) for the questionnaire total scores and for each subscale. Corrected Welch *F* statistics were used because variance differed significantly between groups for all subscales except Vineland-II Communication subscale. In order to provide clinically relevant rough estimates of the rates of common psychological disorders, the numbers of individuals with clinically significant symptoms (*T*-score > 69) reported on the CASI-4R were compared between all four groups and between 22q11.2DS and 22q11.2DupS groups with a chi-square test of independence and Fisher’s exact test when cells contained fewer than five individuals.

In a second set of questionnaire analyses, the 22q11.2DupS group who received a clinician-administered ASD evaluation was subdivided into those with and without a gold standard diagnosis of ASD (“DupASD” *n* = 5; “DupNoASD” *n* = 14; 1 of the 20 individuals in the 22q11.2DupS group who received an ASD evaluation did not complete questionnaires and was not included in this second set of analyses). The DupASD and DupNoASD subgroups were compared to each other and to the idiopathic ASD group and TDC groups, respectively, on the same set of dependent variables as the first set of analyses.

For all analyses, post hoc comparisons were adjusted for multiple comparisons; a Games-Howell test was used in the case of unequal variances and a Tukey’s HSD test when equal variances could be assumed. IBM SPSS Version 22 statistical software was used for all analyses [18].

### Medical and developmental history data collection

#### Participants

This arm of the current study was not restricted by applicable questionnaire age and, as such, all eligible 22q11.2DupS records (*n* = 37) were reviewed and then matched on age and sex to 22q11.2DS patients (*n* = 101). Therefore, all 22q11.2DupS participants included in the psychological analyses were also included in the medical review along with adults with 22q11.2DupS (*n* = 7) and those who declined to complete the psychiatric measures (*n* = 2; see Fig. 1). Resultantly, patients with 22q11.2DupS ranged from ages 2.9 to 46.7 years (mean = 14.0, *SD* = 13.5), while patients with 22q11.2DS ranged from ages 2.0 to 51.1 years (mean = 8.9, *SD* = 7.9). The difference in mean group age reflects the higher rate of parent to child transmission in 22q11.2DupS, which enabled inclusion of more parents of duplication probands in the study.

#### Methods

Data for each participant were collected from a medical and developmental history questionnaire completed by the parent (if available) and from a detailed and systematic review of the participant’s medical record completed by a physician with specialty training in clinical genetics. Adult participants filled out their own medical and developmental history questionnaires. Primary records were not available for seven adult participants who received care outside of our hospital, all of whom were adults identified due to family history of 22q11.2DupS. In these cases, participant self-report of history was used for presence or absence of features. The indication for genetic testing was recorded from the testing requisition and confirmed in medical progress notes whenever possible. A physician confirmed key components noted in the medical and developmental questionnaires through review of clinic notes, progress reports, radiology and laboratory reports, etc. The presence or absence of radiologic testing or evaluation by different subspecialists was documented and radiological reports were reviewed. When questions arose on the review of records, the family was contacted by telephone and discrepancies or questions were addressed.

## Results

### Diagnostic classification and dimensional characterization of ASD in 22q11.2DupS

A review of records of all 22q11.2DupS participants (*n* = 37) revealed that 38 % (*n* = 14) had a community diagnosis of ASD.

**Fig. 2:**
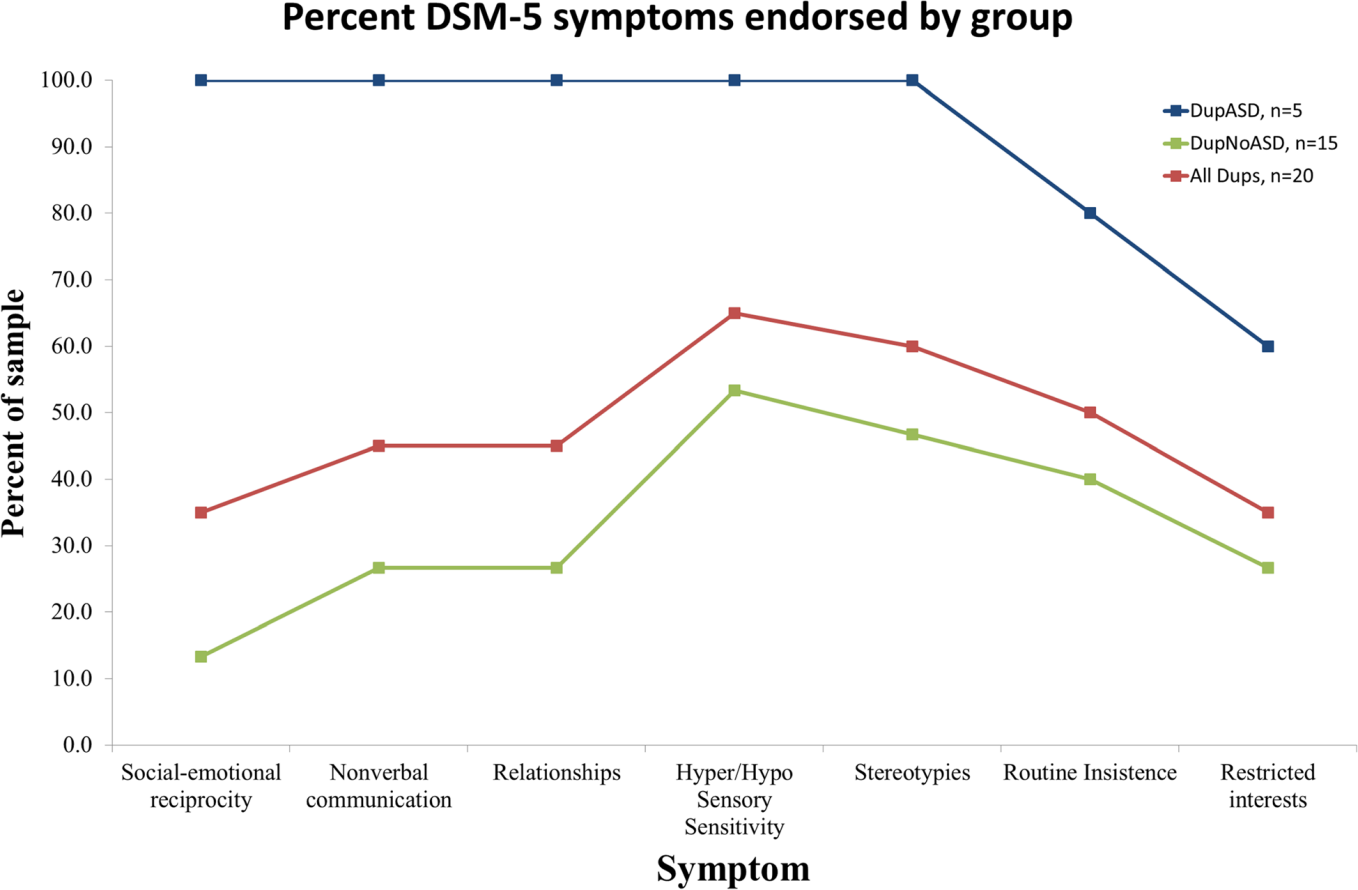
Comparison of autistic symptom distribution in children with 22q11.2DupS, subgrouped by ASD diagnosis. Percentage of the with 22q11.2DupS sample (*Y*-axis) that met individual DSM-5 symptom criteria ASD by expert clinician integration of all of data (ADI-R, ADOS, questionnaires, other clinical observations). The *X*-axis portrays the seven different symptom clusters in DSM-5. Data are portrayed separately for three groupings of 22q11.2DupS: no ASD diagnosis, community ASD diagnosis, and research ASD diagnosis. Those with a community diagnosis include the subset with a research diagnosis. The lower percentages for the sample carrying a community diagnoses might reflect less specific implementation of diagnostic criteria, implementation of older DSM-IV criteria, or improvements in the child’s behavior since the community diagnosis was made. Note: The first three symptoms listed (i.e., difficulties with social-emotional reciprocity, nonverbal communication, and developing relationships) are all required for a DSM-5 diagnosis of ASD; thus, all participants diagnosed with ASD have, by definition, all three of these symptoms. Abbreviations: *ADI-R* Autism Diagnostic Interview-Revised, *ADOS* Autism Diagnostic Observation Schedule, *ASD* autism spectrum disorder

Of the 24 22q11.2DupS participants between 4 and 18 years, we evaluated 20 in this study (12 with the ADI-R only and eight with both the ADI-R and ADOS; Fig. 1). Five of these 20 participants (25 %) met criteria for ASD by consensus clinical judgment based on all of the available data. Of the remaining 15 patients (75 %), many had complex histories and met at least some criteria for an ASD diagnosis. On average, this group had elevated (but not above threshold) scores on the ADI-R and/or ADOS (see Fig. 2).

It should be noted that seven of the 15 participants who did not meet strict research criteria for ASD were previously diagnosed with ASD in a community setting. Clinical presentations were complex, and our best clinical judgment (based on gold standard measures) did not always concur with the community diagnoses. On clinician-completed DSM-5 checklists, these individuals often met criteria in the domain of restricted and repetitive behaviors but not in persistent impairments in social communication and social interactions (see Fig. 2).

Eight participants did not meet research criteria for ASD and had never received a previous diagnosis of ASD. On DSM checklists this group generally showed no or perhaps one symptom (e.g., two children were reported to have sensory aversions and 1 child displayed hand mannerisms). Two children with complex histories showed several symptoms but not enough to meet full ASD criteria by either DSM-IV or DSM-5 criteria.

We also considered whether these 15 participants would meet criteria for social pragmatic communication disorder (SPCD). SPCD is a new diagnosis in DSM-5 and may help describe children with social communication impairments who do not meet full criteria for ASD. We applied DSM-5 SPCD criteria to the detailed behavioral descriptions gathered during the ADI-R and ADOS. None of the 15 participants met the full diagnostic criteria for SCPD, which focuses on pragmatic communication impairments in greetings, sharing information, conversation, understanding nonliteral language, and an ability to modify speech to different social contexts. For most participants, there was clear evidence of good skills in at least some of these areas. The three participants who came closest to SPCD criteria (meeting three of the four behavioral criteria), also showed two or more impairments in the ASD restricted and repetitive behaviors category, thus meeting the restricted behavior criteria for ASD, but not the social communication criteria for either ASD or SPCD.

### Characterization of neuropsychiatric and adaptive behavior profiles for 22q11.2DupS

An analysis of variance (ANOVA) of 22q11.2DS, 22q11.2DupS, ASD, and TDC groups showed significant group differences on total SCQ score (*F*(3, 60.9) = 254.8, *p* < 0.001), total SRS score (*F*(3, 80.9) = 153.3, *p* < 0.001), and Vineland-II Adaptive Behavior Composite score (*F*(3, 84.6) = 80.8, *p* < 0.001) (see Table 2). Post hoc comparisons adjusted for unequal variances revealed significant differences and large effect sizes between the 22q11.2DupS and TDC groups on all measures of social interaction and adaptive behavior skills (SRS *d =* 2.47, 95 % CI_d_ = 1.90–3.03, *p* < 0.001; SCQ: *d =* 2.44, 95 % CI_d_ = 1.78–3.09, *p* < 0.001; Vineland-II Socialization subscale: *d* = 1.50, 95 % CI_d_ = .97–2.03, *p <* 0.001; Vineland-II Adaptive Behavior Composite: *d =* −1.63, 95 % CI_d_ = −2.17 to −1.09, *p* < 0.001). The 22q11.2DS and ASD groups were also significantly more impaired than the TDC group across all measures (*p* < 0.01; see Table 2).

**Table 2:** Group results for psychiatric measurements

	SCQ	SRS	Vineland-II				CASI-4R composite totals					
	Total	Total	Communication	Daily Living	Socialization	Composite	ADHD	ASD	Schizo-affective	Behavior regulation	Depressive	Anxiety
ASD												
mean	21.5^cd^	76.6^cd^	83.9^d^	82.3^d^	74.0^bcd^	78.0^bcd^	13.7^d^	13.2^cd^	3.3^d^	4.8^d^	2.3^d^	4.4^d^
SD	5.3	15.1	14.5	13.5	12.4	11.6	4.1	6.3	1.9	3.4	2.6	2.9
22q11.2DupS												
mean	15.8^d^	69.8^d^	90.6^d^	93.5^d^	90.2^ad^	88.8^ad^	12.9^d^	8.3^d^	2.8	4.3	1.8^d^	3.8^d^
SD	10.9	19.8	17.8	21.6	21.0	19.1	5.9	8.2	1.6	3.8	2.1	3.3
22q11.2DS												
mean	11.3^ad^	66.6^ad^	89.8^d^	88.1^d^	90.2^ad^	87.0^ad^	12.4^d^	6.0^ad^	2.3^d^	4.6^d^	2.2^d^	4.6^d^
SD	7.5	14.9	18.3	15.1	16.8	16.1	5.4	4.9	1.3	2.7	2.8	2.7
TDC												
mean	1.7^abc^	41.9^abc^	113.1^abc^	110.8^abc^	114.0^abc^	113.1^abc^	3.9^abc^	0.3^abc^	0.2^ac^	1.9^ac^	0.3^abc^	0.9^abc^
SD	1.8	5.4	12.8	12.8	12.7	12.4	33.6	0.7	0.3	2.0	0.6	1.2

Measures: SCQ: Raw scores reported. Scores above 15 are strongly suggestive of ASD. SRS: Scores reported in *T*-scores with mean 50 and SD 10. Scores below 60 considered in normal range. CASI-4R: Total raw score for each symptom domain (created by averaging together related subdomains; see Supplemental materials for individual subscale mean scores). Vineland-II: Standard scores (mean 100 and SD 15); scores above 90 considered in the average range. Significantly different (*p* < 0.05) scores between groups on each measure are denoted by the following conventions:

^a^This value is significantly different from the ASD group’s value

^b^This value is significantly different from the 22q11.2DupS group’s value

^c^This value is significantly different from the 22q11.2DS group’s value

^d^This value is significantly different from the TDC group’s value

*Abbreviations*: *ADHD* attention-deficit/hyperactivity disorder, *ASD* autism spectrum disorder, *CASI-4R* Child and Adolescent Symptom Inventory-4R, *SCQ* Social Communication Questionnaire, *SRS* Social Responsiveness Scale, *TDC* typically developing controls, *Vineland-II* Vineland Adaptive Behavior Scales-II

In adjusted post hoc comparisons, the 22q11.2DupS group was less impaired than the ASD group on the Vineland-II Adaptive Behavior Composite (*d = −*0.75, 95 % CI_d_ = −0.26 to −1.24, *p* = 0.048) and the Vineland-II Socialization subscale (*d = −*1.03, 95 % CI_d_ = −0.53 to −1.53, *p* = 0.004), but not on the Daily Living and Communication subscales (see Table 2). However, these results could be driven by the 22q11.2DupS individuals with ASD, since individuals with ASD often show impairment in adaptive skills. Analyses were repeated excluding participants in the 22q11.2DupS group who received a gold standard ASD diagnosis (*n* = 5). Effect sizes became larger (differences ranged from 0.16 to 0.22) but did not change significantly. The duplication group (including individuals with ASD) did not differ from the ASD group on social communication symptoms (SRS *d* = 0.41, 95 % CI_d_ = −0.04 to 0.86, *p* = 0.37; and SCQ *d* = 0.80, 95 % CI_d_ = 0.27 to 1.33, *p* = 0.12), but these results mask significant heterogeneity within the duplications (see below).

**Fig. 3:**
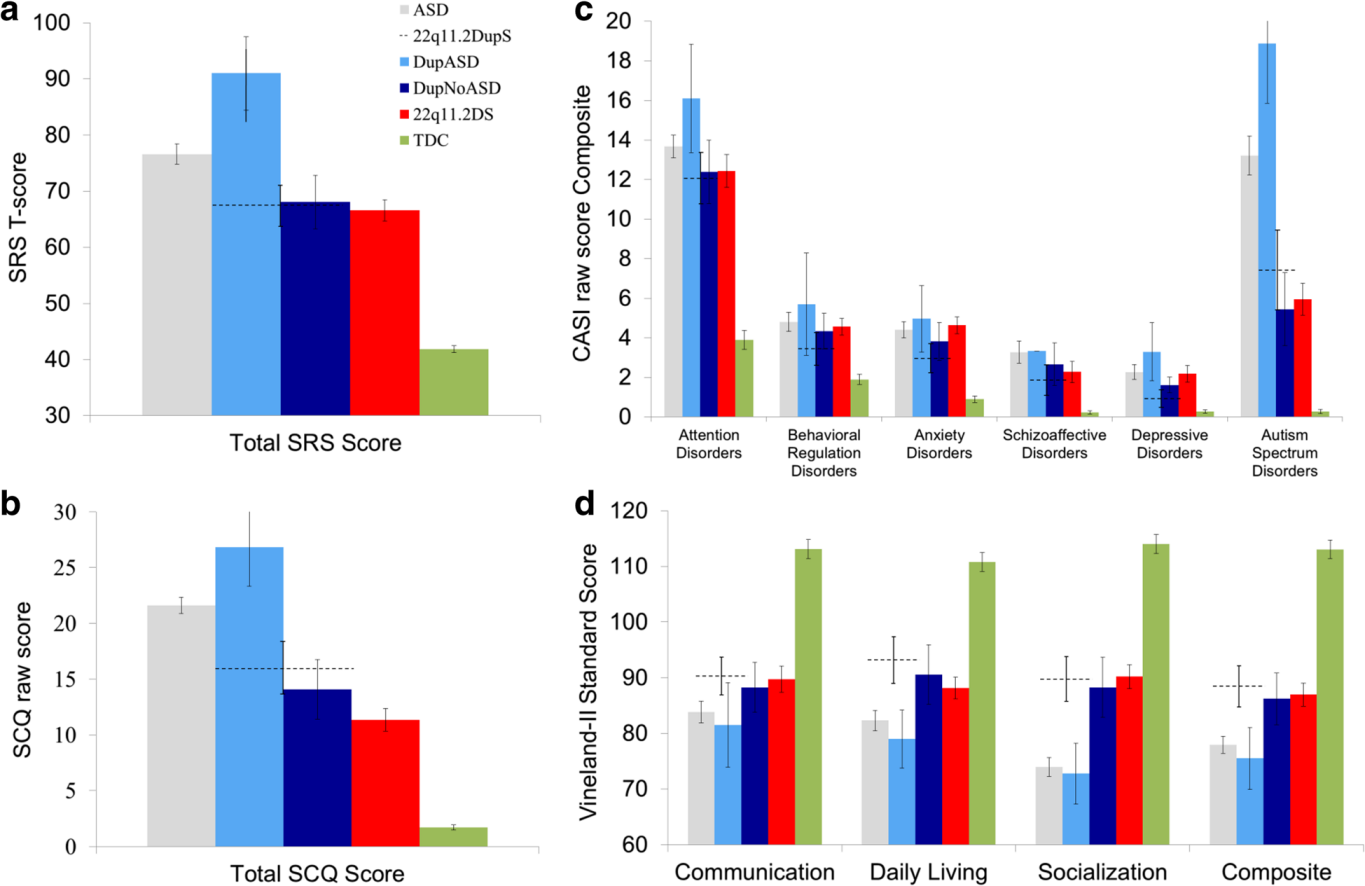
Questionnaire results for participants with 22q11.2DupS, 22q11.2DupS and comorbid ASD, idiopathic ASD, and typical development. Individuals with idiopathic ASD (*gray*), 22q11.2DS (*red*), and TDC (*green*) were compared to individuals with 22q11.2DupS (*dashed line*) on four parent-report questionnaires about behavioral symptoms. *Error bars* represent one standard error. The 22q11.2DupS group was further divided into individuals who have received a gold standard diagnosis of ASD (DupASD; *light blue*) and those who did not (DupNoASD; *dark blue*). On all measures, individuals with 22q11.2DupS showed scores similar to individuals with 22q11.2DS. However, when the 22q11.2DupS group was divided into subgroups, individuals in the DupASD subgroup showed scores similar to individuals with idiopathic ASD, whereas individuals in the DupNoASD subgroup showed mean scores in the average ranges, demonstrating less impairment than individuals with 22q11.2DS. Measures: **a** SRS and **b** CASI-4R: Scores reported in *T*-scores with mean 50 and SD 10. Scores below 60 considered in normal range. **c** SCQ: Raw scores reported. Scores above 15 are strongly suggestive of ASD. **d** Vineland-II: Scores reported in standard scores with mean 100 and SD 15. Scores above 90 considered in the average range. Abbreviations: ASD, autism spectrum disorder; CASI-4R, Child and Adolescent Symptom Inventory-4R; SCQ, Social Communication Questionnaire; SRS, Social Responsiveness Scale; TDC, typically developing children; Vineland-II, Vineland Adaptive Behavior Scales-II

The 22q11.2.DS group was also less impaired than the ASD group on the Vineland-II Adaptive Behavior Composite (*d =* −0.64, 95 % CI_d_ = −1.02 to −0.26, *p* < 0.001) and Vineland-II Socialization subscale (*d = −*1.09, 95 % CI_d_ = −1.49 to −0.69, *p* < 0.001), but like the duplication group, there were no differences in Daily Living and Communication subscales (see Table 2). However, in contrast to the duplication group, the deletion group was significantly less impaired than the ASD group in social communication symptoms (SRS *d* = −0.67, 95 % CI_d_ = −1.03 to −0.31, *p* = 0.001; and SCQ *d* = −1.59, 95 % CI_d_ = −2.03 to −1.15 *p* < 0.001). However, as shown in Table 1, the sample size was much larger for the deletions vs. the duplications, providing more statistical power to find group differences. No significant differences were observed between the 22q11.2DupS and 22q11.2DS groups on any total score (Table 2 and Fig. 3).

The 22q11.2DupS group was subdivided into participants who had received a gold standard diagnosis of ASD (“DupASD,” *n* = 5) and those who did not (“DupNoASD,” *n* = 14). These two subgroups were compared to each other; the DupASD group was also compared to the idiopathic ASD group, and the DupNoASD group to the TDC group (see Table 3 and Fig. 3). The DupASD group showed scores similar to the idiopathic ASD group on measures of social communication and adaptive behavior skills, with no significant differences on any measure of socialization (all *p* > 0.09). The DupNoASD group showed significantly and substantially less social communication impairment than the DupASD group on two of three measures of socialization (SRS *d* = −1.34, 95 % CI_d_ = −2.61 to −0.07, *p =* .02; SCQ *d* = −1.38, 95 % CI_d_ = −2.68 to −0.07, *p =* 0.018; Vineland-II Socialization *d* = −0.82, 95 % CI_d_ = −2.14 to 0.50, *p =* 0.07). However, the DupNoASD group still showed significant impairment when compared to the TDC across all measures, with large effect sizes (all *p* < 0.01). Thus, when this sample of individuals with 22q11.2DupS is taken as an entire group, the group finding of social communication impairment masks important within-group differences associated with the presence or absence of ASD characteristics.

**Table 3:** Comparison of questionnaire data for participants with 22qDupNoASD, 22qDupASD, and idiopathic ASD

	SCQ Total	SRS total	Vineland-II socialization	Vineland-II composite
TDC				
mean (SD)	1.7^a^ (1.8)	41.9^a^ (5.4)	114.0^a^ (12.7)	113.1^a^ (12.4)
* n*	57	73	57	57
DupNoASD				
mean (SD)	14.1^ab^ (9.7)	68.1^ab^ (17.9)	88.3^a^ (20.2)	86.2^a^ (17.3)
* n*	13	14	14	14
DupASD				
mean (SD)	26.8^b^ (7.8)	91.0^b^ (14.6)	72.8 (10.9)	75.5 (11.2)
* n*	5	5	4	4
ASD				
mean (SD)	21.6 (5.3)	76.6 (15.1)	73.9 (12.4)	77.9 (11.6)
* n*	56	68	55	55

Significantly different scores (*p* < 0.05) between groups on each measure denoted as follows:

^a^This value is significantly different between the DupNoASD and Typically Developing Control groups

^b^This value is significantly different between the DupNoASD and DupASD groups

*Abbreviations*: *ADHD* attention-deficit/hyperactivity disorder, *ADI-R* Autism Diagnostic Interview-Revised, *ADOS* Autism Diagnostic Observation Schedule, *ASD* autism spectrum disorder, *CASI-4R* Child and Adolescent Symptom Inventory-4R, *SCQ* Social Communication Questionnaire, *SRS* Social Responsiveness Scale, *TDC* typically developing controls, *Vineland-II* Vineland Adaptive Behavior Scales-II

Next, symptom levels of common psychiatric disorders were compared in 22q11.2DupS, 22q11.2DS, ASD, and TDC groups. Composite scores for six clusters of disorders were computed by averaging raw scores across symptom domains (e.g., dysthymia and major depression were averaged into a “Depression” composite). We used raw scores instead of *T*-scores because we encountered a strong ceiling effect when using CASI-4R *T*-scores because its norms collapse all high raw scores into a *T*-score of 70. The CASI-4R norms that convert raw scores to *T*-scores based on sex and age are useful for comparing raw scores across different ages and sex; however, since our sample groups are matched on sex and age, comparing raw scores does not introduce bias. Adjusted ANOVAs on composite raw scores indicated significant differences between groups in every symptom domain (all *p*’s < 0.001). One of the motivating hypotheses for conducting this study was that we would find lower levels of ADHD and Schizoaffective symptoms in 22q11.2DupS compared to 22q11.2DS. However, post-hoc comparisons revealed no significant differences between these groups in ADHD symptoms, schizoaffective symptoms, nor in any domain of symptoms (all *p*’s > 0.69). All three clinical groups had significantly more ADHD, ASD, depressive, and anxiety symptoms compared to the TDC group (see Fig. 3 and Table 2; effect sizes ranging from −1.03 to −2.94, and *p*’s < 0.01). The 22q11.2DS and the ASD groups (but not the 22q11.2DupS group) were rated as having significantly more schizoaffective and behavioral regulation symptomatology compared to the TDCs (effect sizes ranging from −1.06 to −2.70, and *p*’s < 0.01). Finally, as expected, parent-rated ASD symptoms on the CASI-4R indicated more ASD-related impairments in the ASD group than the 22q11.2DS group (*d =* −1.28, 95 % CI_d_ = −0.77 to −1.78, *p* < 0.05).

We also characterized the rates of clinically significant symptoms across groups. Using a categorical approach, we identified participants with *T*-scores on the CASI-4R in the clinically elevated range (*T*-score > 69). We followed the same domain approach described in the continuous analysis above, such that a participant with clinically elevated symptoms of dysthymia was coded as having clinically elevated symptoms in the depression domain. Next, we compared the prevalence of clinically elevated symptoms across domains between the four groups (TDC, ASD, 22q11.2DS, and 22q11.2DupS) using chi-square tests and two-tailed Fisher’s exact tests when multiple contingency table cells contained fewer than five individuals (schizoaffective, behavioral regulation, and depression domains). As with the analysis of continuous raw scores described above, we observed significant differences in prevalence at the group level in every domain (all *p’*s < 0.05), but no significant differences in prevalence between the duplication and deletion groups (all *p’*s > 0.19) except in the anxiety domain (*χ*^2^(1, *N =* 57) = 4.67, *p* = 0.03), where the deletion group was more impaired. See Additional files 1 and 2.

### Indications for genetic testing within the 22q11.2DupS group

Within the 22q11.2DupS group, the original indications for genetic testing fell in four broad categories—medical issues (*n* = 12, 32 %, one of whom also had a family history), family history (*n* = 10, 27 %), both medical and developmental concerns (*n* = 9, 24 %), and developmental concerns alone (*n* = 6, 16 %). Medical concerns included the following conditions: congenital heart disease, seizures, hypocalcemia, hypotonia, movement disorder, leg length discrepancy/hemihypertrophy, craniosynostosis, cleft palate, hydronephrosis, and hearing loss. Twelve 22q11.2DupS participants had referral for testing because of developmental concerns but no structural birth defect (32 %).

In comparison, across the 22q11.2DS sample indications were as follows: medical issues (*n* = 71; 70 %, one of whom also had a family history), both medical and developmental concerns (*n* = 14; 14 %), developmental concerns (*n* = 9; 9 %), and family history (*n* = 7; 7 %). Medical indications for testing included congenital heart disease, hypocalcemia, feeding difficulties, hypoparathyroidism, postaxial polydactyly, velopharyngeal insufficiency, short stature, cleft palate, bifid thumbs, myelomeningocele, microcephaly, absent thymus, thrombocytopenia, dysmorphic features, clubfeet, laryngeal web, and renal anomalies. A chi-square test of independence showed significant differences in the referral reasons between 22q11.2DupS and 22q11.2DS *χ*^2^ (3, *N* = 138) = 18.44, *p* < 0.001.

Individuals tested for 22q11.2DS due to medical issues alone were generally younger at the time of diagnosis (mean age = 4 months), likely due to medical issues that are apparent early in life, compared to those tested for 22q11.2DupS, who showed fewer medical issues that would be apparent early in life. Individuals with 22q11.2DupS had a mean age of diagnosis of 4.5 years. A review of all available parental testing (22q11.2DupS, *n* = 27; 22q11.2DS, *n* = 91) showed that 22q11.2DupS was inherited from a parent in 66.7 % of patients, compared to 16.5 % of matched 22q11.2DS patients.

### Medical comorbidities

Individuals with 22q11.2DupS had a similar spectrum of birth defects and medical problems as age- and sex-matched individuals with 22q11.2DS, but at lower rates (see Table 4). The rates of medical comorbidities in our 22q11.2DS sample were similar to those reported in the literature. Rates of these conditions in 22q11.2DupS are not available for comparison; however, the types of medical problems in our cohort of 22q11.2DupS patients are similar to prior published cases.

**Table 4:** Medical problems in 37 patients with 22q11.2DupS compared to 101 patients with 22q11.2DS

System	22q11.2DupS with documentation of evaluation	Percent abnormal of those with evaluation	Comments	22q11.2DS with documentation of evaluation; Percent abnormal
Cardiac	Clinical evaluation: *n* = 37	24 % (9/37)	VSD, PVS, PDA, ASD/PFO, TOF, HLHS	Clinical evaluation by echo: *n* = 99; 82 % abnormal
	Echo: *n* = 25			
Endocrine	Endocrine evaluation: *n* = 31	Hypothyroidism 19 % (6/31) Hypocalcemia 10 % (3/31)		Endocrine evaluation: *n* = 98; 70 % abnormal (including 16 % with hypothyroidism and 38 % with hypocalcemia)
Hearing loss	Audiogram: *n* = 37	16 % (6/37) • Conductive *n* = 3 • Mixed *n* = 2 • Sensorineural *n* = 1	Mild loss in 4/5; Mild to moderate loss in 1/5 with Mixed HL	Audiogram: *n* = 101; 34 % abnormal (including 26 % with Conductive HL, 3 % with sensorineural; 2 % with HL not specified)
Hematologic	Complete blood count: *n* = 37	16 % (6/37) • Thrombocytopenia *n* = 1 • Thrombocytopenia and anemia *n* = 1 • Neutropenia *n* = 2 • Anemia n = 2	Prolonged PTT in patient with Factor XII deficiency not included	Complete blood count: *n* = 101; 23 % abnormal (including 12 % with thrombocytopenia)
Immunologic	Immunology visit: *n* = 31	39 % (12/31) • Abnormal immunoglobulin levels *n* = 7 • Inappropriate vaccine response *n* = 3 • Low T-cell count *n* = 1 • CVID n = 1		Immunology visit: *n* = 98; 66 % abnormal
Neurologic/calavarium	Brain MRI: *n* = 16	Structural anomaly 24 % (5/21)	Chiari Type I; Prominent posterior CSF space, platybasia, T2 prolongation in hippocampus; Sagittal synostosis	Neurological evaluation: *n* = 50; 40 % abnormal (including 15 % with seizure disorder)
	EEG: *n* = 5	Hypotonia 27 % (10/37)		
		Seizure activity 19 % (7/37)		
Ophthalmologic	Ophthalmology evaluation: *n* = 37	22 % (8/37) • Strabismus *n* = 5 • Megalocornea *n* = 1 • Aphakia and congenital cataract *n* = 1 • Ptosis *n* = 1		Ophthalmology evaluation: *n* = 56; 48 % abnormal (including 23 % with strabismus)
Otolaryngology(non-palate related)	Otolaryngology evaluation: *n* = 37	54 % (20/37)	Ankyloglossia, dysphagia, laryngomalacia, Eustachian tube dysfunction. Surgical interventions: BMT (*n* = 9), T&A (*n* = 9)	Otolaryngology evaluation: *n* = 93; 83 % abnormal (including 59 % requiring BMTs and 20 % requiring T&A)
Renal	Renal ultrasound: *n* = 25	24 % (6/25)	VUR; Pelviectasis (*n* = 2); Lithiasis; Nephromegaly; Megaureter	Renal ultrasound: *n* = 95; 23 % abnormal
Skeletal	C-Spine x-rays: *n* = 11	C-spine anomaly 45 % (5/11)	Slightly large atlantodens interval; Hypoplastic PE of C1 and elongated PE of C2; Exaggerated kyphosis, lordosis; Incomplete arch C1; Lack of bony fusion of C1 and dysmorphic C2	C-Spine x-ray: *n* = 59; 71 % abnormal (27 % with C2-C3 fusion). Hemihypertrophy noted in 1/101.
		Hemihypertophy noted in 3/37		

Participants: Medical problems listed by system that were observed in 37 previously unreported patients with 22q11.2DupS compared to 101 patients with 22q11.2DS. 22q11.2DS patients were matched on age and sex to the 22q11.2DupS patients. Note: not all diagnoses are listed and those listed are may not be mutually exclusive

*Abbreviations*: *ASD/PFO* atrial septal defect/patent foramen ovale, *BMT* bilateral myringotomy tubes, *CSF* cerebrospinal fluid, *CVID* common variable immunodeficiency, *Echo* echocardiogram, *EEG* electroencephalogram, *HL* hearing loss, *HLHS* hypoplastic left heart syndrome, *MRI* magnetic resonance imaging, *PDA* patent ductus arteriosus, *PE* posterior elements, *PTT* partial thromboplastin time, *PVS* pulmonary valve stenosis, *T&A* tonsillectomy with adenoidectomy, *TOF* tetralogy of Fallot, *VSD* ventricular septal defect, *VUR* vesicoureteral reflux

### Utility of medical screening in patients tested for developmental concerns or family history

Although there are no published guidelines for screening in 22q11.2DupS, it is customary for all patients with 22q11.2DupS seen by our “22q and You” clinic to receive the same set of medical screening evaluations and tests as patients with 22q11.2DS [2]. This allowed us the unique opportunity to determine the utility of this screening, especially in patients referred for developmental concerns or because of an affected family member. These evaluations uncovered 20 previously unsuspected medical problems, strongly supporting the use of screening guidelines developed for 22q11.2DS in 22q11.2DupS (see Table 5). Table 5 is based on the 12 patients for whom genetic testing was requested with an indication of developmental delay or autism without a known somatic birth defect. We did not exclude patients from this category for medical problems common in children with developmental issues such as hypotonia. However, it is possible that there were birth defects that were known but not indicated at the time of testing. The rate of medical comorbidities detectable by screening remained high when only selecting children who were known to have autism or developmental delay but no reported birth defect. Most of the types of medical problems detected in Table 5 would not be easily detected without a targeted test (e.g., inadequate response to vaccines, mild to moderate hearing loss, hypothyroidism, cervical spine anomalies).

**Table 5:** Medical findings in 22q11.2DupS patients because of genetic testing ordered without indication of birth defect

Abnormalities by system	Rate	Comments
Endocrine	1/12	Hypothyroidism *n =* 1
Gastrointestinal	2/12	Hepatomegaly *n* = 1
		Splenomegaly *n* = 1
Hearing loss • Conductive • Mixed conductive/sensorineural • Sensorineural	4/12 • 2/12 • 1/12 • 1/12	Mild *n* = 2 Mild *n* = 1 Mild *n* = 1
Hematologic	3/12	Thrombocytopenia *n* = 1
		Neutropenia *n* = 2
Immunologic	5/12	Low pneumococcal titers following vaccine *n* = 2
		Common variable immunodeficiency *n* = 1
		Low tetanus antibodies *n* = 1
		Dysgammaglobulinemia *n* = 1
Palate	2/12	Velopharyngeal insufficiency *n* = 1
		Bifid uvula^a^ *n* = 1
Spine	3/12	Large atlantodens interval with platybasia *n* = 1
		Scoliosis *n* = 1
		Incomplete arch of C2 *n* = 1

Medical problems indicated in the table are the resultant findings from 12 patients with previously undiagnosed 22q11.2DupS after genetic testing was ordered with an indication of developmental delay or autism. To our knowledge, none of the 12 patients had a previously known somatic birth defect, however, it is possible that birth defects were known to the ordering physician but not indicated at the time of testing. Most medical problems noted here would not have been easily detected without a targeted test (e.g., inadequate response to vaccines, mild to moderate hearing loss, hypothyroidism, cervical spine anomalies)

^a^Not a medical problem in isolation but is an anomaly of the palate that is sometimes associated with palatal dysfunction or unrecognized submucous cleft palate, so is indicated here

## Discussion

Over a third of our sample of individuals (38 %) with confirmed 22q11.2DupS received a community diagnosis of ASD. However, further evaluation by clinicians working full time in autism specialty clinics using gold standard instruments suggested the true rate of ASD in 22q11.2DupS was less, with a conservative estimate between 14 % (5 of 37 with record review) and 25 % (5 of 20 research-evaluated). Another third of our sample showed significant and heterogeneous developmental issues that overlap with ASD features without meeting full diagnostic criteria. No participants met criteria for social pragmatic communication disorder (SPCD). This is the first study, to our knowledge, to apply gold standard research testing for ASD to the 22q11.2DupS population. A first estimate that 14–25 % of patients with 22q11.2DupS have ASD is much higher than population prevalence estimates of 1–2 % [19], and higher than comparable data for the deletion syndrome.

The rate of confirmed ASD in 22q11.2DupS in this study (14–25 %) is among the highest of any genetic syndrome associated with ASD, and the rate of community diagnoses of ASD (38 %) is certainly among the highest. In the absence of an epidemiological sample, the absolute rate of ASD in different disorders must be interpreted with caution and attention to the sampling and assessment methods that were used to determine the prevalence. Our sample includes individuals who were referred for SNP microarray testing for any reason, and does not include individuals unknowingly carrying the 22q11.2 duplication without an indication for SNP microarray testing; thus, our reported prevalence rate is most relevant to samples of individuals with known 22q11.2DupS. The upper range of our prevalence estimate (5/20) corresponds to the rate of ASD among individuals who elected to participate in an ADI. However, only 5 of 25 eligible individuals declined, so the potential overestimation bias is quite small (5 %). The lower range of our reported prevalence estimate (5/37) is conservative. Therefore, 14–25 % represents an accurate range of ASD prevalence in samples of children with known 22q11.2DupS.

Children with 22q11.2DupS who showed characteristics of ASD but did not meet full research criteria often presented with symptoms in the domain of restricted and repetitive behaviors, while failing to meet formal criteria for social communication impairments. This pattern contrasts with the symptom pattern reported in a study of 100 individuals with 22q11.2DS [3] where more social communication deficits were identified than restricted and repetitive interests [3]. If this difference between 22q deletions and duplications is found to be reliable in future studies, it suggests that individuals with deletion and duplication may have a “reciprocal” phenotype that would allow for complementary study of the two primary symptom clusters found in those with ASD. Many of these patients received a community diagnosis of ASD prior to study enrollment. Several had rich imaginary worlds that occupied a large amount of the child’s free time, and seemed somewhat atypical because of the repetitive quality and often excessive amounts of time spent with imaginary daydreaming. Again, if our findings are replicated by others the 22q genetic region may impact these areas of development in a dose-sensitive manner.

Prior research has shown that children with 22q11.2DS have a high rate of medical comorbidities. Because of the known risk of occult medical problems with high rate of morbidity and mortality, screening guidelines have been proposed for 22q11.2DS. Children with 22q11.2DS are also more commonly diagnosed because of structural birth defects by providers (e.g., neonatologists, geneticists, cardiologists) familiar with the need for rather extensive medical screening. No clear guidelines exist for 22q11.2DupS. The utility of performing the extensive medical screening that is done in 22q11.2DS on children with developmental issues alone, in the absence of any known medical issues, has not been previously assessed. Moreover, the medical screening for 22q11.2DS is extensive and costly.

Our clinical practice has been to perform all 22q11.2DS-related screening in patients diagnosed with 22q11.2DupS. This provided us with an opportunity to assess retrospectively whether that screening of this population resulted in a meaningful identification of occult medical problems. Screening resulted in identification of 20 individual medical problems among the 12 individuals with 22q11.2DupS where genetic testing was ordered with an indication of developmental delay or autism with no previously known medical diagnoses. Many of these would have no obvious medical symptoms but pose significant risk for morbidity and/or mortality, including cervical spine anomalies, inadequate response to vaccines, and hypothyroidism. Additional medical problems, such as identification of mild to moderate hearing loss, could lead to difficulties in school without appropriate intervention. They also all require a targeted test to diagnose, which is included in the medical screening guidelines. Several medical problems were identified during screening of individuals with 22q11.2DupS where the indication for testing was isolated developmental issues, ASD or an affected family member. This suggests that the medical screening should be done in all cases of 22q11.2DupS, regardless of indication for testing or presence of known medical problems at the time of diagnosis.

The spectrum of medical problems was similar in 22q11.2DS and 22q11.2DupS but the rate of comorbidities was lower in 22q11.2DupS. We conclude that the medical screening guidelines for 22q11.2DS should be applied to the 22q11.2DupS population to minimize morbidity and mortality related to occult medical problems. Microarray testing has become increasingly utilized in individuals with developmental delay and ASD, which is supported by the American College of Medical Genetics. Due to this increase in genetic testing of children with ASD, increasing numbers of children with ASD are being identified with microdeletion and microduplication syndromes, including 22q11.2DupS. The rate of medical problems in Table 4 is likely to be accurate for structural anomalies that do not change with age, but may be an underestimate for medical conditions (e.g., hypothyroidism, hypocalcemia), as additional patients may develop these conditions over time.

Newly detected medical problems in patients referred for developmental concerns, rather than existing medical problems, is an important clinical finding. Psychiatrists, developmental pediatricians, and general pediatricians will likely identify the majority of children with 22q11.2DupS while following recommended guidelines for genetic testing in ASD. These practitioners should be aware of the need for children with 22q11.2DupS to undergo medical screening including echocardiogram, renal ultrasound, cervical spine x-rays, immunologic testing (or referral to immunologist for evaluation), ionized calcium levels, thyroid functioning, and audiologic evaluation. Given the elevated rate of ophthalmologic abnormalities in our cohort, an ophthalmologist should also evaluate them. It should be emphasized that these recommendations are based on our entire cohort of children with 22q11.2DupS as noted in Table 4. The medical problems identified on screening in children with no previously known medical problems, as noted in Table 5, suggest that the same screening should be applied to all patients with 22q11.2DupS regardless of the types of medical problems that are known at the time of diagnosis.

As all children in this study received the same screening, it is impossible to perform a retrospective comparison of medical problems that would have resulted from missed medical problems. However, the types of medical problems that occurred in our population are known to result in significant morbidity (e.g., undetected structural heart disease can cause sudden death, untreated hypothyroidism can cause intellectual disability, children with cervical spine anomalies are at greater risk for spinal cord injuries with hyperextension of the neck and may need special techniques if undergoing anesthesia and be restricted from certain sports, undetected renal malformations can place the child at risk for ascending urinary tract infections and kidney damage, children with some immunodeficiencies should not receive live vaccinations and may require additional doses of some vaccines to produce immunity). Providers caring for children diagnosed with 22q11.2DupS should consider these children vulnerable to the medical problems in Table 4, which often are asymptomatic until irreversible damage has been done. Therefore, screening for these conditions in all children diagnosed with 22q11.2DupS should be done.

In our study, we found that children with 22q11.2DS were typically identified at an earlier age due to birth defects and serious medical problems. This is similar to other reports in the literature [20] and we also found that children with congenital heart disease had earlier diagnoses than children without congenital heart disease. While these children went on to develop more slowly than their peers and had neuropscychiatric comorbidities, their genetic diagnosis was usually already known. In contrast, children with 22q11.2DupS were typically diagnosed because of their neuropsychiatric and developmental differences with subsequent identification of medical comorbidities.

As the first study to our knowledge to assess rigorously both medical conditions and ASD symptoms in individuals with 22q11.2DupS, we note several directions for future research. First, future studies should examine the relationship (or lack of relationship) between ASD characteristics and specific medical conditions. Second, the symptom presentation and developmental trajectories of patients with both ASD and 22q11.2DupS may have some similarities and some differences when compared to either idiopathic ASD or other syndromic forms of ASD. Third, the mean age of our samples was about 8 years, and thus information about ASD symptoms during later stages of development could reveal differences from what was found here. Fourth, the high rate of gold standard confirmed ASD in this population suggests that animal models of 22q11.2DupS may be more informative models of ASD than 22q11.2DS or other genetic models, although as noted above, it is possible that deletions and duplications will model different parts of overall portrait of ASD. All of these lines of work may help elucidate underlying biological mechanisms at work in ASDs from different etiologies.

Genetic syndromes associated with higher rates of ASD may better help us tease apart ASD characteristics, even if they cannot help us understand the most common presentation of ASD, which is idiopathic. With a rate of about 25 % with ASD, and another third with developmental issues, the fact remains that a significant number of individuals with 22q11.2DupS show no signs of ASD or related developmental concerns. Additional study the of the mechanisms underlying 22q11.2-related syndromes may uncover precise reasons for those that have increased risk of ASD, as well as the factors that protect against ASD and other developmental delays in the context of gene dosage effects in this region of chromosome 22.

## Conclusions

This is the first prospective study of a cohort of individuals with 22q11.2DupS. The rate of ASD in 22q11.2DupS in our cohort was 14–25 %, with an additional third of patients showing some features of ASD or other neuropsychiatric concerns. Our results provide support for microarray testing in children with idiopathic ASD, as prospective management would be altered for children identified to have 22q11.2DupS. Our 22q11.2DupS sample exhibited similar types of medical problems as individuals with 22q11.DS, but at lower rates. Thus, our data also suggests all individuals who are diagnosed with 22q11.2DupS should receive medical screening for 22q11.2-associated medical conditions. Finally, as the rate of ASD in 22q11.2DupS is among the highest of any genetic syndrome with a low rate of comorbid medical conditions, 22q11.2DupS may be an excellent candidate for future research into syndromic models of ASD.

## Additional files

Additional file 1: Table S1. Psychiatric symptoms measured by CASI-4R in participants with idiopathic ASD, 22q11.2DupS, 22q11.2DS, and typical development. (DOC 47 kb) Additional file 2: Table S2. Proportion of cohorts with *T*-scores in the ‘high’ range for psychiatric symptoms measured by CASI-4R. (DOC 39 kb)

## Abbreviations

22q11.2DS: 22q11.2 deletion syndrome
22q11.2DupS: 22q11.2 duplication syndrome
ADHD: attention-deficit/hyperactivity disorder
ADI-R: autism diagnostic interview-revised
ADOS: Autism Diagnostic Observation Schedule
ASD: autism spectrum disorder
CASI-4R: Child and Adolescent Symptom Inventory-4R
CHOP: Children’s Hospital of Philadelphia
DSM: diagnostic and statistical manual of mental disorders
SCQ: Social Communication Questionnaire
SRS: Social Responsiveness Scale
TDC: typically developing children
Vineland-II: Vineland Adaptive Behavior Scales-II
