# Erratum to: 22q11.2 duplication syndrome: elevated rate of autism spectrum disorder and need for medical screening

**DOI:** 10.1186/s13229-016-0097-5

**Published:** 2016-07-13

**Authors:** Tara L. Wenger, Judith S. Miller, Lauren M. DePolo, Ashley B. de Marchena, Caitlin C. Clements, Beverly S. Emanuel, Elaine H. Zackai, Donna M. McDonald-McGinn, Robert T. Schultz

**Affiliations:** Department of Pediatrics, Seattle Children’s Hospital, M/S OB.9.5204800 Sand Point Way NE, Seattle, WA USA; Center for Autism Research, Children’s Hospital of Philadelphia, 3535 Market Street, Philadelphia, PA 19104 USA; Department of Psychology, University of Pennsylvania, 3720 Walnut Street, Philadelphia, PA 19104 USA; Division of Human Genetics and Molecular Biology, Children’s Hospital of Philadelphia, 3401 Civic Center Boulevard, Philadelphia, PA 19104 USA; 22q and You Center, Children’s Hospital of Philadelphia, 3401 Civic Center Boulevard, Philadelphia, PA 19104 USA; Department of Pediatrics, University of Pennsylvania, 3401 Civic Center Boulevard, Philadelphia, PA 19104 USA

## Erratum

After publication of the research article [1], the authors noticed a minor error in panel **d** of Fig. 3. The authors would like to draw the reader’s attention towards the corrected Fig. 3 below. The corrected panel **d** reflects a very minor change which does not change the overall conclusion of the study.

**Fig. 3:**
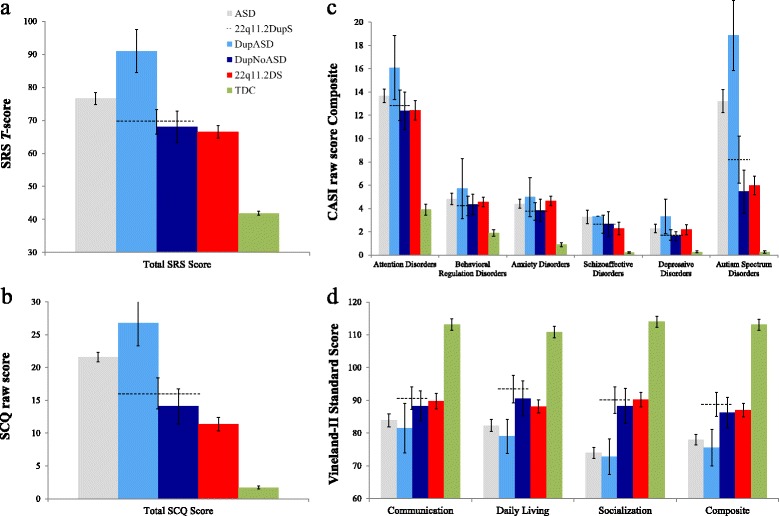
Questionnaire results for participants with 22q11.2DupS, 22q11.2DupS and comorbid ASD, idiopathic ASD, and typical development. Individuals with idiopathic ASD (*gray*), 22q11.2DS (*red*), and TDC (*green*) were compared to individuals with 22q11.2DupS (*dashed line*) on four parent-report questionnaires about behavioral symptoms. *Error bars* represent one standard error. The 22q11.2DupS group was further divided into individuals who have received a gold standard diagnosis of ASD (DupASD; *light blue*) and those who did not (DupNoASD; *dark blue*). On all measures, individuals with 22q11.2DupS showed scores similar to individuals with 22q11.2DS. However, when the 22q11.2DupS group was divided into subgroups, individuals in the DupASD subgroup showed scores similar to individuals with idiopathic ASD, whereas individuals in the DupNoASD subgroup showed mean scores in the average ranges, demonstrating less impairment than individuals with 22q11.2DS. Measures: **a** SRS and **b** CASI-4R: Scores reported in T-scores with mean 50 and SD 10. Scores below 60 considered in normal range. **c** SCQ: Raw scores reported. Scores above 15 are strongly suggestive of ASD. **d** Vineland-II: Scores reported in standard scores with mean 100 and SD 15. Scores above 90 considered in the average range. Abbreviations: ASD, autism spectrum disorder; CASI-4R, Child and Adolescent Symptom Inventory-4R; SCQ, Social Communication Questionnaire; SRS, Social Responsiveness Scale; TDC, typically developing children; Vineland-II, Vineland Adaptive Behavior Scales-II

Figure 3: This figure shows the corrected Fig. 3 of the original article.
