# Supplementary material for: 22q11.2 duplication syndrome: elevated rate of autism spectrum disorder and need for medical screening
Source: Mol Autism. 2016 May 6;7:27. doi: 10.1186/s13229-016-0090-z (PMC4859984; doi:10.1186/s13229-016-0090-z)
Supplement: Additional file 1: Table S1. — Psychiatric symptoms measured by CASI-4R in participants with idiopathic ASD, 22q11.2DupS, 22q11.2DS, and typical development. (DOC 47 kb) [file 13229_2016_90_MOESM1_ESM.doc]

**Additional file 1: Table S1.** Psychiatric symptoms measured by CASI-4R in participants with idiopathic ASD, 22q11.2DupS, 22q11.2DS, and typical development.

|  |  | Inattention | Hyperactivity | ADHD combined | ODD | CD | GAD | Social Phobia | Separation Anxiety | Schizoid | Schizophrenia | Depression | Dysthymia | ASD | Asperger’s | Bipolar |
| --- | --- | --- | --- | --- | --- | --- | --- | --- | --- | --- | --- | --- | --- | --- | --- | --- |
| ASD | n | 52 | 52 | 52 | 52 | 52 | 52 | 41 | 52 | 11 | 11 | 52 | 52 | 41 | 41 | 11 |
|  | mean | 15.7 | 11.6 | 27.4 | 8.3 | 1.3 | 6.9 | 3.1 | 2.6 | 3.6 | 2.6 | 2.1 | 2.4 | 16 | 10.5 | 3.5 |
|  | SD | 5.3 | 4.8 | 8.3 | 5.3 | 2.3 | 4.2 | 2.4 | 3.6 | 2.2 | 2 | 2.4 | 3 | 7.7 | 5.1 | 4.1 |
| Dup | n | 21 | 21 | 21 | 21 | 21 | 21 | 17 | 21 | 4 | 4 | 21 | 21 | 17 | 17 | 4 |
|  | mean | 13.8 | 12 | 25.8 | 7 | 1.6 | 5.9 | 1.9 | 3.1 | 1.5 | 2 | 1.8 | 1.9 | 9.8 | 6.8 | 5 |
|  | SD | 5.1 | 7.9 | 11.7 | 5.6 | 2.7 | 4.1 | 2.7 | 3.7 | 1.3 | 2.2 | 2.5 | 1.9 | 9.7 | 6.8 | 3.4 |
| Del | n | 43 | 43 | 43 | 43 | 43 | 43 | 37 | 43 | 6 | 6 | 43 | 43 | 37 | 37 | 6 |
|  | mean | 14.5 | 10.3 | 24.9 | 8 | 1.1 | 7.3 | 2.9 | 3.7 | 3 | 0.8 | 2 | 2.4 | 7.1 | 4.8 | 3 |
|  | SD | 5.6 | 6.3 | 10.9 | 4.3 | 1.5 | 4.6 | 2.3 | 3.7 | 1.3 | 0.8 | 2.7 | 2.9 | 5.6 | 4.2 | 3 |
| TDC | n | 57 | 57 | 57 | 57 | 57 | 57 | 45 | 57 | 12 | 12 | 57 | 57 | 45 | 45 | 12 |
|  | mean | 4.7 | 3.1 | 7.8 | 3.6 | 0.2 | 1.8 | 0.2 | 0.6 | 0.3 | 0 | 0.2 | 0.3 | 0.3 | 0.2 | 0.3 |
|  | SD | 4 | 4.3 | 7.2 | 3.5 | 0.8 | 2.3 | 0.7 | 1.3 | 0.5 | 0 | 0.5 | 0.7 | 0.8 | 0.6 | 0.7 |

Raw scores on each symptom subscale of the CASI-4R.

Abbreviations: ADHD, attention-deficit/hyperactivity disorder; ASD, autism spectrum disorder; CASI-4R, Child and Adolescent Symptom Inventory-4R; CD, conduct disorder; Del, 22q11.2DS; Dup, 22q11.2DupS; GAD, generalized anxiety disorder; ODD, oppositional defiant disorder; TDC, typically developing children.
