# Supplementary material for: 22q11.2 duplication syndrome: elevated rate of autism spectrum disorder and need for medical screening
Source: Mol Autism. 2016 May 6;7:27. doi: 10.1186/s13229-016-0090-z (PMC4859984; doi:10.1186/s13229-016-0090-z)
Supplement: Additional file 2: Table S2. — Proportion of cohorts with T-scores in the ‘high’ range for psychiatric symptoms measured by CASI-4R. (DOC 39 kb) [file 13229_2016_90_MOESM2_ESM.doc]

**Additional file 2: Table S2.** Proportion of cohorts with *T*-scores in the ‘high’ range for psychiatric symptoms measured by CASI-4R.

|  |  | Inattention | Hyperactivity | ADHD combined | ODD | CD | GAD | Social Phobia | Separation Anxiety | Schizoid | Schizophrenia | Depression | Dysthymia | ASD | Asperger’s | Bipolar |
| --- | --- | --- | --- | --- | --- | --- | --- | --- | --- | --- | --- | --- | --- | --- | --- | --- |
| ASD | n | 52 | 52 | 52 | 52 | 52 | 52 | 41 | 52 | 11 | 11 | 52 | 52 | 41 | 41 | 11 |
|  | % | 50 | 36.5 | 57.7 | 19.2 | 7.7 | 28.8 | 31.7 | 21.2 | 45.5 | 36.4 | 7.7 | 17.3 | 87.8 | 73.2 | 9.1 |
| Dup | n | 21 | 21 | 21 | 21 | 21 | 21 | 17 | 21 | 4 | 4 | 21 | 21 | 17 | 17 | 4 |
|  | % | 42.9 | 47.6 | 47.6 | 9.5 | 9.5 | 28.6 | 17.6 | 23.8 | 0 | 25 | 4.8 | 4.8 | 47.1 | 47.1 | 0 |
| Del | n | 43 | 43 | 43 | 43 | 43 | 43 | 37 | 43 | 6 | 6 | 43 | 43 | 37 | 37 | 6 |
|  | % | 41.9 | 34.9 | 37.2 | 7 | 2.3 | 34.9 | 37.8 | 18.6 | 33.3 | 0 | 9.3 | 23.3 | 43.2 | 32.4 | 0 |
| TDC | n | 57 | 57 | 57 | 57 | 57 | 57 | 45 | 57 | 12 | 12 | 57 | 57 | 45 | 45 | 12 |
|  | % | 0 | 3.5 | 0 | 1.8 | 0 | 3.5 | 0 | 1.8 | 0 | 0 | 0 | 0 | 0 | 0 | 0 |

The proportion of participants with idiopathic ASD, 22q11.2DupS, 22q11.2DS, and TDCs who showed *T*-scores in the ‘high’ range for various psychiatric disorders based on parent report on the CASI-4R. *T*-scores in the ‘high’ range (>69) suggest clinically significant levels of symptoms.

Abbreviations: ADHD, attention-deficit/hyperactivity disorder; ASD, autism spectrum disorder; CASI-4R, Child and Adolescent Symptom Inventory-4R; CD, conduct disorder; Del, 22q11.2DS; Dup, 22q11.2DupS; GAD, generalized anxiety disorder; ODD, oppositional defiant disorder; TDC, typically developing children.
